# Metagenomic analysis of bacterial species in tongue microbiome of current and never smokers

**DOI:** 10.1038/s41522-020-0121-6

**Published:** 2020-03-13

**Authors:** Noriaki Sato, Masanori Kakuta, Takanori Hasegawa, Rui Yamaguchi, Eiichiro Uchino, Wataru Kobayashi, Kaori Sawada, Yoshihiro Tamura, Itoyo Tokuda, Koichi Murashita, Shigeyuki Nakaji, Seiya Imoto, Motoko Yanagita, Yasushi Okuno

**Affiliations:** 1 Department of Biomedical Data Intelligence, Graduate School of Medicine, Kyoto University, 54 Shogoin Kawahara-cho, Sakyo-ku, Kyoto, 606-8507 Japan; 2 Department of Nephrology, Graduate School of Medicine, Kyoto University, 54 Shogoin Kawahara-cho, Sakyo-ku, Kyoto, 606-8507 Japan; 3 Human Genome Center, The Institute of Medical Science, The University of Tokyo, 4-6-1 Shirokanedai, Minato-ku, Tokyo, 108-8639 Japan; 4 Health Intelligence Center, The Institute of Medical Science, The University of Tokyo, 4-6-1 Shirokanedai, Minato-ku, Tokyo, 108-8639 Japan; 5 Department of Medical Intelligent Systems, Graduate School of Medicine, Kyoto University, 54 Shogoin Kawahara-cho, Sakyo-ku, Kyoto, 606-8507 Japan; 6 Department of Oral and Maxillofacial Surgery, Hirosaki University Graduate School of Medicine, 5 Zaifu-cho Hirosaki, Aomori, 036-8562 Japan; 7 Department of Social Medicine, Hirosaki University Graduate School of Medicine, 5 Zaifu-cho Hirosaki, Aomori, 036-8562 Japan; 8 Department of Oral Health Care, Hirosaki University Graduate School of Medicine, 5 Zaifu-cho Hirosaki, Aomori, 036-8562 Japan; 9 COI Research Initiatives Organization, 5 Zaifu-cho Hirosaki, Aomori, 036-8562 Japan

**Keywords:** Microbial genetics, Plaque, Next-generation sequencing

## Abstract

Cigarette smoking affects the oral microbiome, which is related to various systemic diseases. While studies that investigated the relationship between smoking and the oral microbiome by 16S rRNA amplicon sequencing have been performed, investigations involving metagenomic sequences are rare. We investigated the bacterial species composition in the tongue microbiome, as well as single-nucleotide variant (SNV) profiles and gene content of these species, in never and current smokers by utilizing metagenomic sequences. Among 234 never smokers and 52 current smokers, beta diversity, as assessed by weighted UniFrac measure, differed between never and current smokers (pseudo-F = 8.44, *R*^2^ = 0.028, *p* = 0.001). Among the 26 species that had sufficient coverage, the SNV profiles of *Actinomyces graevenitzii*, *Megasphaera micronuciformis*, *Rothia mucilaginosa*, *Veillonella dispar*, and one *Veillonella sp.* were significantly different between never and current smokers. Analysis of gene and pathway content revealed that genes related to the lipopolysaccharide biosynthesis pathway in *Veillonella dispar* were present more frequently in current smokers. We found that species-level tongue microbiome differed between never and current smokers, and 5 species from never and current smokers likely harbor different strains, as suggested by the difference in SNV frequency.

## Introduction

Cigarette smoking is associated with many oral diseases such as periodontitis and oral cancer^1^. The oral microbiome are reported to play a vital role in the pathogenesis of such oral diseases^2^, and are also important in systemic diseases such as diabetes mellitus^3^ and rheumatoid arthritis^4^. The change of oral microbiome induced by cigarette smoking could be associated with systemic diseases; thus, the relationship between cigarette smoking and the oral microbiome is of interest.

Recently, numerous studies investigating quantitative differences in oral microbiome across smoking status using 16S rRNA amplicon sequencing technology have been performed. An analysis of mouth rinse samples performed by Kato et al., revealed that *Neisseria* was less abundant, while members of the *Veillonellaceae* family were more abundant, in current smokers^5^. A large study, conducted by Wu et al., indicated that anaerobic bacteria favor the oral environment in smokers, and that aerobic bacteria were less abundant^6^. Moreover, they revealed that the functional metagenomic profile, which included the degradation of certain toxic compounds contained in cigarettes, also differed between current and never smokers. Mason et al., investigated differences in the subgingival community and also identified elevation of anaerobic bacteria and cariogenic species^7^. However, although species-level differences in diversity and abundance were investigated using 16S rRNA amplicon sequencing in some previous studies, investigations involving metagenomic sequences are rare, as previous studies suggested that species-level prediction based on 16S rRNA had low accuracy^8^. Besides, there are few studies that investigated differences in single-nucleotide variant (SNV) profiles and gene content per species in the oral environment across smoking status, which cannot be explored by amplicon sequencing.

Here, we conducted the metagenomic profiling of tongue plaque samples in a Japanese health-checkup cohort. Species-level differences of diversity and abundance, as well as SNV profiles and gene content of the bacterial species, were investigated across the smoking status.

## Results

### Characteristics and statistics of participant metagenomic sequences

**Fig. 1:**
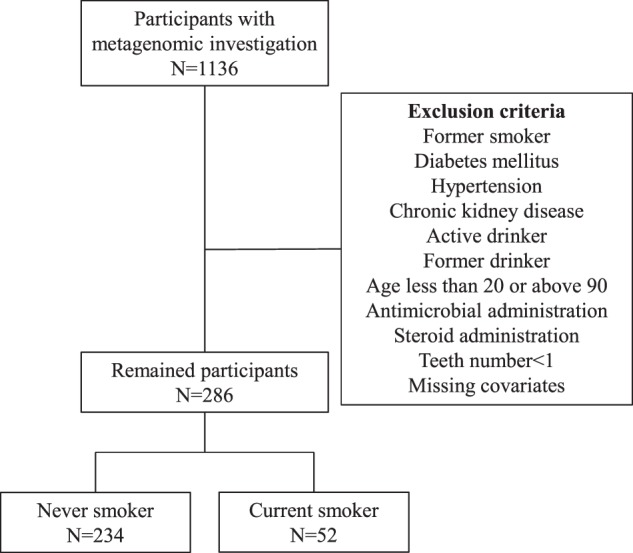
Flowchart detailing participant selection. Flowchart detailing participant selection. Overall, 286 participants were selected.

**Fig. 2:**
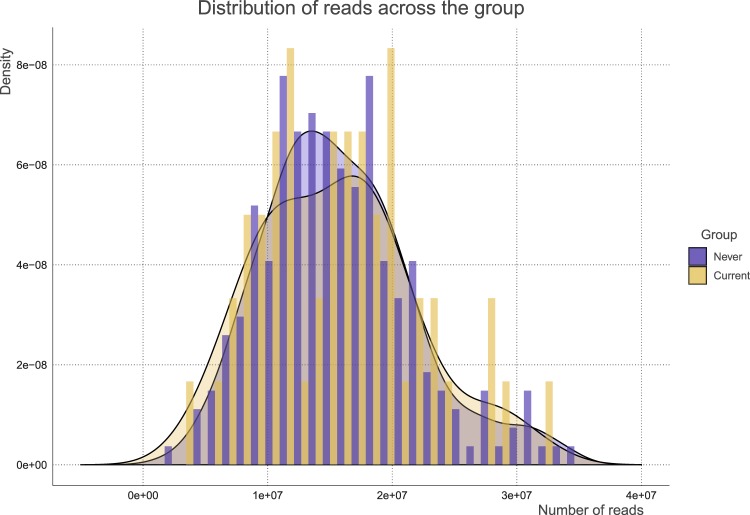
The distribution of reads across groups. The barplot and distribution of reads across groups of never and current smokers are shown. Blue indicates never smokers, while yellow indicates current smokers.

A flowchart summarizing participant selection for this study is shown in Fig. 1. Table 1 details the background of the 286 participants included in the study. There were significant differences in sex, BMI category, and presence of caries between current and never smokers. After quality filtering and removal of human genome contamination, the average reads (SD) across participants was 9769630 (3920068) and the minimum and maximum numbers of reads were 3169504 and 24023935, respectively. The distribution of filtered reads across the groups were visualized in Fig. 2.

**Table 1:** Participants’ background.

Clinical values	Never smokers (*n* = 234)	Current smokers (*n* = 52)	*p*-value
Age (years), mean (SD)	47.99 (15.47)	43.56 (10.53)	0.05
Sex: # female (%)	192 (82.1)	32 (61.5)	0.002
Teeth number, mean (SD)	25.63 (5.55)	26.15 (5.30)	0.534
eGFR (mL/min/1.73 m^2^), mean (SD)	82.59 (15.15)	83.76 (12.03)	0.605
HbA1c (%), mean (SD)	5.66 (0.30)	5.68 (0.29)	0.675
Systolic blood pressure (mmHg), mean (SD)	115.09 (11.94)	111.94 (12.04)	0.087
Diastolic blood pressure (mmHg), mean (SD)	70.11 (8.53)	68.81 (10.13)	0.337
Pack-year index, mean (SD)	NaN (NA)	13.77 (15.13)	NA
FEV1.0%, mean (SD)	83.28 (6.54)	82.06 (6.73)	0.228
BMI category # (%)			0.003
0–18.5 kg/m^2^	23 (9.8)	10 (19.2)	
≥18.5–25 kg/m^2^	173 (73.9)	28 (53.8)	
≥25–30 kg/m^2^	29 (12.4)	14 (26.9)	
≥30	9 (3.8)	0 (0.0)	
Caries present # (%)	66 (28.2)	23 (44.2)	0.036
Suspected of having periodontitis # (%)	117 (50.0)	33 (63.5)	0.109

*BMI* body mass index, *HbA1c* hemoglobin A1c, *eGFR* estimated glomerular filtration rate, *FEV1.0%* percent predicted forced expiratory volume in 1 s, *SD* standard deviation.

### Differences in beta diversity and relative abundance

**Fig. 3:**
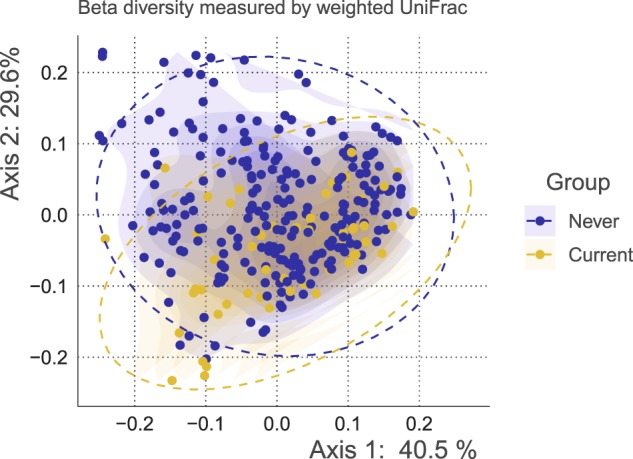
The principal coordinate plot of beta diversity measured by weighted UniFrac. The x- and y-axes represent the first and second principal coordinates with the proportion of variance. The 95% confidence ellipse is shown for each group. The points in blue indicate never smokers, while the points in yellow indicate current smokers.

**Fig. 4:**
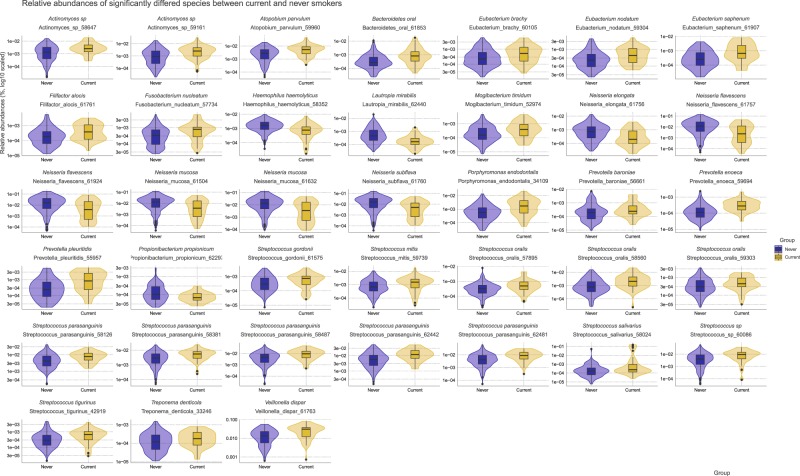
The violin and boxplot demonstrating significant differences in relative species abundance. The group of never or current smokers are shown on the x-axis and the relative abundances is shown on the y-axis (on a logarithmic scale). Blue indicates abundance in never smokers, while yellow indicates abundance in current smokers.

First, beta diversity and relative abundance were compared to characterize overall microbiome differences at the species-level. Beta diversity was compared between groups by weighted UniFrac measure, and a statistically significant difference were identified (pseudo-F = 8.44, *R*^2^ = 0.028, *p* = 0.001). The PCoA plot of weighted UniFrac distance is shown in Fig. 3. Next, the relative abundance of the species was compared. Overall, 1157 species were present across all samples. Among them, 313 species that have a relative abundance of at least 0.0001 in above 20% of all the samples were tested by MaAsLin2, and 38 species showed statistically significant differences in relative abundance. We visualized the violin and box plot of relative abundances for these species (Fig. 4). 29 species including *Porphyromonas endodontalis* (coef. = 0.023, standard error = 0.0037, *q* < 0.001), *Streptococcus oralis* (coef. = 0.020, standard error = 0.0041, *q* < 0.001), *Streptococcus parasanguinis* (coef. = 0.036, standard error = 0.0071, *q* < 0.001) were significantly present more abundant in current smokers, while nine species including *Neisseria subflava* (coef. = −0.062, standard error = 0.013, *q* = 0.0012), *Lautropia mirabilis* (coef. = −0.013, standard error = 0.0031, *q* = 0.0027), *Neisseria flavescens* (coef. = −0.050, standard error = 0.013, *q* = 0.0087) were present less abundant in current smokers. Species were listed according to the lowest 3 *q*-values. All the statistical results are presented in Supplementary Data Set 1. Additionally, species relative abundance table were deposited in Supplementary Data.

### Differences in SNV frequency

**Fig. 5:**
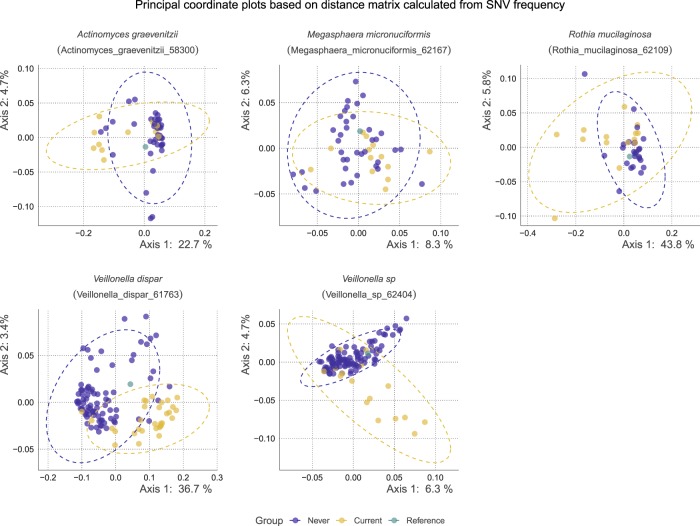
The principal coordinate plots based on the distance matrix of species differed significantly between never and current smokers in terms of SNV frequency. The x- and y-axes represent the first and second principal coordinates, respectively, with the proportion of variance. The points in blue indicate never smokers, while the points in yellow indicate current smokers, and the green points indicate the reference in the database.

**Fig. 6:**
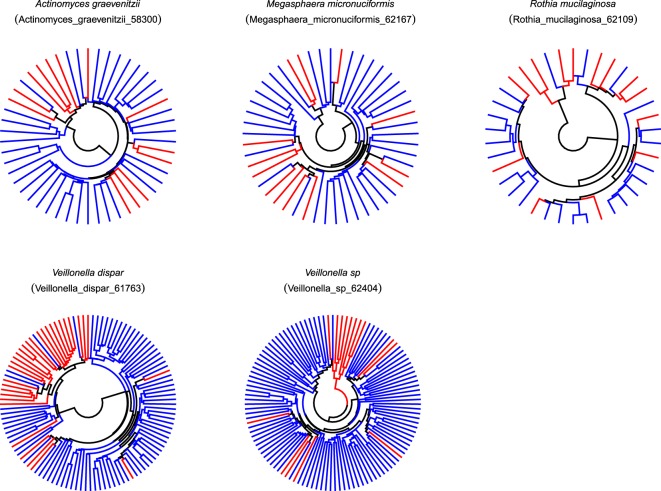
The dendrogram based on the distance matrix of species that differed significantly between never and current smokers in terms of SNV frequency. The circular dendrogram is shown for each species significantly differed in terms of SNV frequency. Red branches indicate the leaf of current smokers, and blue branches indicate the leaf of never smokers.

We next analyzed whether there are differences in bacterial genome SNV frequency across smoking status. For SNV profiling, 26 species that have sufficient coverage in at least 10% of the samples were analyzed. The number of participants with sufficient read depth for the species is summarized in Supplementary Data Set 1. Among these 26 species, a distance matrix calculated from the SNV frequency table was compared by permutational multivariate analysis of variance (PERMANOVA). Overall, 5 species; *Actinomyces graevenitzii* (profiled in 38 never and 13 current smokers, pseudo-F = 2.60, *R*^2^ = 0.051, *q* = 0.013, Actinomyces_graevenitzii_58300 in the MIDAS database), *Megasphaera micronuciformis* (profiled in 37 never and 13 current smokers, pseudo-F = 1.56, *R*^2^ = 0.032, *q* = 0.021, Megasphaera_micronuciformis_62167 in the MIDAS database), *Rothia mucilaginosa* (profiled in 25 never and 14 current smokers, pseudo-F = 6.47, *R*^2^ = 0.124, *q* = 0.009, Rothia_mucilaginosa_62109 in the MIDAS database), *Veillonella dispar* (profiled in 81 never and 31 current smokers, pseudo-F = 20.98, *R*^2^ = 0.16, *q* = 0.009, Veillonella_dispar_61763 in the MIDAS database), one *Veillonella sp* (profiled in 116 participants, 100 never and 16 current smokers, pseudo-F = 2.71, *R*^2^ = 0.023, *q* = 0.009, Veillonella_sp_62404 in the MIDAS database) showed statistically significant differences in SNV frequency between current and never smokers. The PCoA plots and dendrograms based on SNV frequency are shown in Figs 5 and 6. Those 5 species were selected for the downstream analysis. The exact number of SNVs and the genome length were summarized in Table 2. Participants’ background with average sufficient read depth of species included in the downstream analysis are summarized in Supplementary Data Set 1. The SNV location and SNV frequency of the samples for all the species are deposited in Supplementary Data. As the proportion of participants with “suspected of periodontitis” was higher in current smokers, we performed the comparison between those with healthy periodontal status and those with suspected of periodontitis controlling for age, sex, BMI, teeth number, caries status, and smoking. There were no species that showed significant differences between them (Supplementary Data Set 1). We performed the analysis with the newly created custom database for the 5 species that significantly differed, and the result was similar (Supplementary Data Set 1). We additionally performed multiple sequence alignment of the core-genome of these 5 species, and visualize the tree in Supplementary Fig. 1. Additionally, the dendrogram based on the distance matrix calculated from SNV frequency table, with RefSeq deposited sequences are visualized in Supplementary Fig. 2. Further, the proportion of within- and between-group strain sharing are described in Supplementary Data Set 1.

**Table 2:** The detailed number of SNVs and the genome length.

Species_id	Number_of_snv	Number_of_snv_in_cds	m-equal-r(count)	m-equal-r(%)	Genome_length
Actinomyces_graevenitzii_58300	97,158	90,456	80,295	0.82643735	2,196,917
Megasphaera_micronuciformis_62167	87,204	80,271	77,932	0.8936746	1,765,528
Rothia_mucilaginosa_62109	299	274	283	0.94648829	2,264,603
Veillonella_dispar_61763	5930	4714	4983	0.84030354	2,118,767
Veillonella_sp_62404	28,233	25,005	25,319	0.89678745	2,176,752

*m-equal-r* the number of SNV that the major allele are as same as the reference allele.

### Differences in gene and pathway content of species

**Fig. 7:**
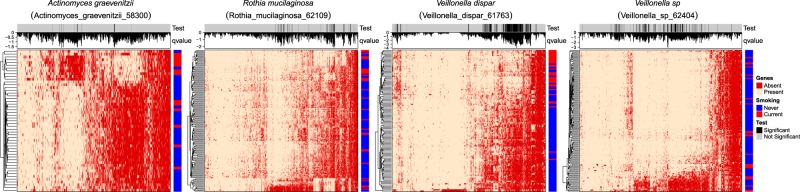
Heatmap depicting gene presence or absence status. Rows indicate sample (red: current smokers, blue: never smokers), and columns indicate statistical significance (gray: not significant, black: significant) along with *q*-values (on a logarithmic scale). Heatmap cells indicate gene presence or absence (red: absent, yellow: present).

We next investigated differences in gene and pathway content across species present in current or never smokers. The gene presence table was analyzed by logistic regression models. The gene differential results for 5 species were summarized in Table 3. There were no significant differences in terms of gene presence frequency between current and never smokers in *Megasphaera micronuciformis*. Those genes yielded up-regulated and down-regulated KO identifiers for each species and the KEGG pathways were inferred by MinPath. Up-regulated pathways were as follows; purine and pyrimidine metabolism, and lipopolysaccharide biosynthesis in *Veillonella dispar*, pentose phosphate pathway, glycine, serine and threonine metabolism, and phenylalanine, tyrosine and tryptophan biosynthesis in *Rothia mucilaginosa*. Down-regulated pathways were as follows; galactose metabolism in *Actinomyces graevenitzii*, pyrimidine metabolism and ubiquinone and other terpenoid-quinone biosynthesis in *Rothia mucilaginosa*, glycolysis/gluconeogenesis, cysteine and methionine metabolism, and pyrimidine metabolism in *Veillonella dispar*, and pentose phosphate pathway, purine metabolism, and cysteine and methionine metabolism in *Veillonella sp*. Note that the pathway with the highest three total number of involved KO identifiers was listed, and genes that were differentially presented between current and never smokers are listed in Supplementary Data Set 1, and the inferred pathways are summarized in Supplementary Data Set 1. In addition, a gene status heatmap displaying presence or absence of four species that have significantly differed genes is shown in Fig. 7. Further, the gene presence table for all the species were deposited in Supplementary Data.

**Table 3:** The summary of the differential analysis of genes.

Species_id	Never	Current	Total-gene	All-zero	All-one	Tested	Down	Up
Actinomyces_graevenitzii_58300	38	13	2471	46	918	1507	10	7
Megasphaera_micronuciformis_62167	37	13	1724	87	1210	427	0	0
Rothia_mucilaginosa_62109	107	30	1765	2	187	1576	24	37
Veillonella_dispar_61763	85	31	1890	10	349	1531	24	271
Veillonella_sp_62404	115	23	1991	19	303	1669	197	3

*Never* the number of never smokers profiled for genes, *Current* the number of current smokers profiled for genes, *Total-gene* the number of total genes, *All-zero* the number of genes that were absent in all samples, *All-one* the number of genes that were present in all samples, *Tested* the number of genes tested, *Down* the number of genes down-regulated, *Up* the number of genes up-regulated.

### Random forest classification

We performed binary classification of current and never smokers by random forest algorithm to characterize how these differences can discriminate current and never smokers. The performance was highest when the input was gene presence table of *Veillonella dispar* (0.930 [0.060], mean [SD]). The performance was 0.913 [0.077], 0.824 [0.098], 0.817 [0.107], and 0.801 [0.128] respectively (mean [SD]), when the input was SNV frequency table of *Veillonella dispar*, SNV frequency table of *Veillonella sp*, gene presence table of *Veillonella sp*, and SNV frequency table of *Actionmyces graevenitzii*. Only the model with AUROC above 0.8 were listed, and other performances regarding 5 species profiled were summarized in Table 4. When the input was the relative abundance of all the species presented in samples, the performance was 0.745 [0.097]. These results indicated that current and never smokers can be discriminated by the SNV frequency or gene presence table of *Veillonella dispar*, one of *Veillonella sp*, and *Actinomyces graevenitzii*, which suggested these species within the oral microbiome of current and never smokers have distinct SNV frequency or gene presence frequency characteristics.

**Table 4:** The performance of random forest classifier measured by AUROC.

Input	Species	Mean	Stdev
relative_abundances	–	0.7453	0.0971
snv	Actinomyces_graevenitzii_58300	0.7396	0.2034
snv	Megasphaera_micronuciformis_62167	0.6631	0.1182
snv	Rothia_mucilaginosa_62109	0.7467	0.204
snv	Veillonella_dispar_61763	0.9132	0.0773
snv	Veillonella_sp_62404	0.8238	0.0984
Gene	Actinomyces_graevenitzii_58300	0.8012	0.1283
Gene	Megasphaera_micronuciformis_62167	0.6554	0.1977
Gene	Rothia_mucilaginosa_62109	0.6842	0.0939
Gene	Veillonella_dispar_61763	0.9298	0.0596
Gene	Veillonella_sp_62404	0.8174	0.1069

*Mean* the mean value of 5-fold cross validation, *Stdev* the standard deviation value of 5-fold cross validation.

## Discussion

This study explored the species-level bacterial composition, SNV profile, and gene content of species in the tongue microbiome, with particular focus on the relationship with cigarette smoking. The tongue is exposed to many chemical compounds during cigarette smoking and many of these compounds are considered harmful^9^. In the present study, we found 5 species that differed in terms of SNV frequency. The relative abundance of *Veillonella dispar* was high in current smokers, and the SNV profile differed in current smokers compared to never smokers, which suggests that current smokers have different strains of *Veillonella dispar*. It seems likely that *Veillonella* genus is related with cigarette smoking, considering the results of previous studies which reported quantitative changes of *Veillonella*^5,6^.

*Veillonella* are strict-anaerobic, Gram-negative diplococci from the phylum Firmicutes that are commensal oral bacteria, and are reported to be nitrate and nitrite reducing bacteria^10^. They were also reported in some clinical case reports such as prosthetic joint infection^11^. In various studies using 16S rRNA amplicon sequencing technology, *Veillonella* were reported to be related to smoking. Wu et al. reported that *Veillonella* were more abundant in mouthwash samples of smokers, and suggested that the anaerobic characteristics of these bacteria make them tolerant to the smoking environment^5^. Kato et al. also found that the *Veillonellaceae* family were more abundant in smoker’s saliva samples^4^. *Veillonellaceae* were also reported to be related to certain cancers, such as lung cancer, and are reported to be a good marker of squamous cell carcinoma in the salivary microbiome^12^. In addition, *Veillonella parvula* were reportedly found frequently in the lower air tracts of lung cancer patients^13^. Furthermore, a study comparing microbiome in bronchoalveolar lavage fluid of lung cancer patients and benign mass patients revealed that *Megasphaera* and *Veillonella* were more abundant in lung cancer patients^14^.

Additionally, *Veillonella dispar* gene and pathway content analysis revealed that some genes in *Veillonella dispar* were present with high frequency in current smokers compared to never smokers. Interestingly, the lipopolysaccharide (LPS) biosynthesis pathway was up-regulated in the current smoker group, from the pathway reconstruction based on the significantly different genes. LPS are located in outer membrane of Gram-negative bacteria like *Veillonella*, and consist of lipid A, core oligosaccharide, and a distal polysaccharide^15^. In the review by Delwiche et al., they postulated that *Veillonella* produces significant endotoxic LPS by sugar assimilation of not only ribose, but also fructose being incorporated into LPS^16^. Additionally, *Veillonella* LPS were reported to have relatively high endotoxicity^17^. Our results suggested that *Veillonella dispar* have a potentially different activities related to LPS production in current and never smokers, judging from the KEGG pathway analysis.

We identified quantitative differences between bacteria in current and never smokers at the species-level. In accordance with other studies, anaerobic bacteria, including those in the genus *Streptococcus* or *Veillonella*, as well as *Fusobacterium nucleatum*, were confirmed to be more abundant in current smokers^7^. In the genus *Veillonella*, *Veillonella dispar* was significantly more abundant in terms of relative abundance. In addition to quantitative differences measured by 16S rRNA sequence, which has also been described in some previous studies, the present study provided more specific information regarding differences in the tongue microbiome between current and never smokers.

Our results suggested that *Veillonella dispar* gene presence table was able to distinguish current smokers and never smokers with the highest performance using the random forest classifier, which indicated that *Veillonella dispar* from current and never smokers have distinct characteristics compared to other species investigated. Additionally, the sequences of strains deposited in RefSeq under *Veillonella dispar* were placed in different groups in the dendrogram, suggesting that strains deposited in RefSeq could be grouped according to other criteria, such as SNV frequency and functional differences.

Further, our study demonstrated that *Actinomyces graevenitzii*, *Megasphaera micronuciformis*, *Rothia mucilaginosa*, and one *Veillonella sp* also displayed the potential of having different strains at the species-level based on the SNV frequency difference, and some genes from those species were significantly differed. *Actinomyces graevenitzii* has not been reported in association with cigarette smoking. *Megasphaera micronuciformis* were reported to be found more commonly in cigarette smoker’s upper gastrointestinal tract, as detected using the microarray^18^, and they are under the family *Veillonellaceae*. The genus *Rothia* is an oral commensal, and *Rothia mucilaginosa* was recently reported to be increased in current smoker’s buccal microbiome^19^, although our analysis in tongue microbiome did not find significant difference in terms of relative abundance. We performed strain sharing pattern analysis to further elucidate the meaning of these differences, and two *Veillonella* species have relatively high strain sharing within groups compared to between groups. However, we cannot extrapolate functional consequences based on these SNV findings. Additionally, there is currently no strict definition of strain^20^, and the definition is given by the tool basis.

The current study has several strengths. The study explored differences in SNV profiles of tongue microbiome across smoking status using community-wide metagenomics data, and it revealed that smokers may harbor different strains in some species compared to never smokers. Further, we profiled the differences in gene content for these species, which had not been thoroughly investigated previously. As well, we minimized the effect of confounders by various parameters which are said to affect oral microbiome.

The present study also contained limitations. First, due to the cross-sectional nature of the study, it is unknown whether these results indicate that species present originally in the tongue flora underwent nucleotides alteration due to cigarette smoking, or whether some species favor the habitat created by smoking. In addition, we did not include dietary information, which may also influence the tongue microbiome. The detailed functional consequences, as well as the clinical implication, of differences in SNV or gene profiles remain unclear and further investigations will be necessary. Online tools other than MIDAS, such as StrainPhlAn^21^ or metaSNV^22^, are available to investigate species or strain-level microbiome profiles. These tools utilize different databases, and differences in databases may influence the profile results. The default database of MIDAS utilized clustering with a cutoff of 95% genome-wide ANI, which correspond to the standard definition of species. Thus, the present analysis could detect variations under the species-level, and we validated the result using a custom-built database based on RefSeq. We cannot determine which species got sufficient read depth to profile SNV beforehand. Additionally, we could not dissect the effect of smoking and periodontitis on the tongue microbiome thoroughly as cigarette smoking is a risk factor for periodontitis, and the prevalence of periodontitis in current smokers in our cohort was high. In addition, the oral examination was based on one-time measurement when they admitted to the health-checkup, so the detailed treatment history of oral diseases, or how often they got maintenance treatment regarding oral diseases were unknown.

In summary, we conclude that tongue microbiome of cigarette smokers differed from that of never smokers at a species-level resolution. Moreover, *Veillonella dispar* was suggested to have a different SNV profile and gene content in smokers, thus implying different functionality. In addition to previous studies that mainly investigated quantitative differences in relative abundances of the oral microbiome, we show that differences related to SNV profile and gene content may also be present between current and never smokers. However, the clinical implication of these differences requires further investigation.

## Methods

### Study population and covariate assessment

This study was approved by the Ethical Committee of Hirosaki University (approval number: 2016-028) and all participants provided written informed consent. The participants were drawn from the Iwaki health-checkup cohort conducted in 2016. The participants’ covariate information was obtained from questionnaires administered upon induction into the study. Medical history, current medication use, and smoking and drinking history was provided by the participants. Body mass index (BMI) was calculated from participants’ height and weight, and was classified into four categories according to the World Health Organization criteria. Smoking status was classified as current, former, and never. Drinking status was classified as non-drinker, current drinker, and former drinker. Natural tooth number, caries number, and periodontal status were directly examined by the dentists, and caries number was categorized as having caries or not. Periodontal status was classified as suspected of having periodontitis or not. Specifically, if participants had the a depth of gingivitis pocket ≥4 mm and/or gum bleeding, they were categorized as suspected of having periodontitis, according to the definition of the Community Periodontal Index^23^. Patients were excluded based on the following criteria: patients younger than 20 years or older than or equal to 90 years, use of antihypertensive drugs,　selection of “currently under treatment”, “currently followed up” or “previously under treatment” of hypertension in the questionnaire, systolic blood pressure of 140 mmHg or above, diastolic blood pressure of 90 mmHg or above, use of antidiabetic drugs, selection of “currently under treatment”, “currently followed up” or “previously under treatment” of diabetes mellitus in the questionnaire, HbA1c of 6.5 or above, estimated glomerular filtration rate below 30, use of antimicrobials or steroids, current or former drinkers, former smokers, those who have no teeth, and those with missing information regarding any of their covariates. Overall, 52 current smokers and 234 never smokers were included in the study. We measured the predicted percentage of forced expiratory volume in 1 s and pack-year index (the number of cigarettes smoked per day divided by 20, multiplied by the number of years of smoking).

### Sample collection, DNA extraction, and Illumina shotgun sequencing

For each of the participants, in the morning of admission and before breakfast and tooth brushing, tongue plaque samples were obtained by brushing the dorsal surface of tongue 4–5 times with a swab. The swab head was then placed in a collection tube containing 4 M guanidium thiocyanate, 100 mM Tris-HCl (pH 8.0), 40 mM EDTA and 0.001% bromothymol blue. The samples were mixed with zirconia beads using a FastPrep 24 instrument (MP Biomedicals, Santa Ana, California, USA). DNA was extracted from the bead-treated suspensions using an automatic nucleic acid extractor and MagDEA DNA 200 (GC) (Precision System Science, Chiba, Japan). Quantification and quality assessment of extracted DNA was performed using the 2200 TapeStation System (Agilent, Santa Clara, California, USA). DNA samples were fragmented using LE220 (Covaris, Woburn, Massachusetts, USA), and subjected to library preparation using the TruSeq ChIP Library Preparation Kit (Illumina, San Diego, California, USA) following the manufacturer’s instructions. Prepared libraries were evaluated using the 2200 TapeStation System, and quantified by quantitative PCR using KAPA Library Quantification Kits (KAPA Biosystems, Wilmington, Massachusetts, USA). Sequencing was performed on the HiSeq2500 instrument (Illumina, San Diego, California, USA) with 101 bp paired-end reads.

### Metagenomic sequence processing

Raw paired-end reads were quality filtered via sickle version 1.33^24^ using the sliding window approach and the following parameters: average quality threshold of 30 and the minimum length of 20. Reads were then mapped to the human reference genome (GRCh38) by Burrows-Wheeler Aligner MEM (BWA-MEM) version 0.7.17^25^ with the default parameter set and mapped reads with alignment length greater than 80 were subsequently discarded.

### Relative abundance and SNV profiling

Metagenomic Intra-Species Diversity Analysis System (MIDAS) was used to profile species abundance, SNV frequency, and gene content in each species^26^. First, we aligned quality-filtered paired-end sequence reads to the reference database, containing 31,007 sequences comprising 5952 species provided by default in MIDAS, by HS-BLASTN^27^ to obtain species-level abundance per sample (run_midas.py species in default parameter, and merge_midas.py species with default parameter). The resulting alignment files were processed by MIDAS to profile SNVs against the bacterial representative genome (run_midas.py snps with the default parameter). Specifically, species with ≥3.0X coverage (total base pair aligned to the representative sequence of the species divided by total genome length of the species) were profiled SNV. Subsequently, the SNV profile was merged across samples, and only bi-allelic positions were chosen. Participants with average read depth across reference sites with at least 1 mapped read of ≥5.0 were merged SNV per species, and species with a number of profiled participants ≥10% of the total number of participants were included in the SNV analysis. Other parameters were in accordance with the preset option ‘–core-snps’ (merge_midas.py snps –core-snps). We additionally performed a validation analysis using the updated custom database for species that differed significantly in terms of SNV frequency. The complete genome sequences for species significantly differed were downloaded from the NCBI RefSeq^28^ based on the assembly summary on 17 December 2019. The average nucleotide identity (ANI) of the sequences was calculated by FastANI^29^, and the sequences were hierarchically clustered based on the ANI. The representative sequence was chosen based on the medoid of the clusters, and the build_db.py script was deployed. We again analyzed the SNV frequency differences for significant species using the newly created database and computed the result. Additionally, the SNV profile of metagenomics in our cohort was compared with that of sequences available in the NCBI RefSeq. In order to compare the reference sequences with our metagenomic sequences, we simulated 9,000,000 metagenomic sequence reads from reference sequences using wgsim, as MIDAS expect metagenomic sequences to profile SNV, with parameters set to simulate no errors, insertions, or deletions. Subsequently, these reads were processed by the MIDAS pipeline via the same method used to profile metagenomic samples from the cohort. Hierarchical clustering based on the distance matrix calculated from SNV frequency was performed and a dendrogram was constructed for visualization of the result.

### Gene content and pathway analysis

We also performed pan-genome gene content analysis to infer the gene content of species per sample by MIDAS using the default parameters (run_midas.py genes with the default parameter), and results were merged across samples (merge_midas.py genes with the default parameter). Gene presence status was visualized by a heatmap, produced by R library ComplexHeatmap^30^. Additionally, we inferred functional pathways using significantly up-regulated or down-regulated genes to compare the functional characteristic of species between groups. Specifically, the nucleotide sequences of significantly differed genes were aligned to the Kyoto Encyclopedia of Genes and Genomes (KEGG) prokaryotic protein database^31^ by DIAMOND version 0.8.22^32^ with the default parameter. One gene was matched to one KEGG prokaryotic protein with the lowest e-value. Subsequently, the protein was matched to KEGG Orthologue (KO) database. The KEGG pathways were inferred by MinPath version 1.4 from the resulting KO identifier. MinPath infers the conservative estimate of pathways by a parsimony approach rather than a naïve mapping approach using a set of genes^33^.

### Statistical analysis

Participant backgrounds were compared via one-way ANOVA and chi-squared test. Beta diversity was assessed by the weighted UniFrac measure, and compared between current and never smokers by PERMANOVA using adonis function in R package vegan^34^, adjusted for age, sex, BMI category, teeth number, presence of caries, and periodontal status, and was subsequently visualized by principal coordinate analysis (PCoA) plot. Default phylogenetic trees provided by MIDAS were used. Beta diversity calculation was performed by the function UniFrac in R library phyloseq^35^. Species abundance was compared between current and never smokers using Multivariate Association with Linear Models 2 (MaAsLin2)^36^ adjusted for age, sex, BMI category, teeth number, presence of caries, and periodontal status. Relative abundances were arc sine square root transformed, and taxa that had a minimum abundance of 0.0001 in at least 20% of all samples were tested (“transform = AST”, “min_abundance = 0.0001”, and “min_prevalence = 0.2”). The distance between samples was calculated based on the Manhattan distance, which accounted for the sum of the differences of non-reference allele frequency per SNV positions divided by the number of SNV positions. These distances were calculated based on the sub-set of participants that have sufficient average read depth for the candidate species. Subsequently, these distances were tested for association with smoking status by PERMANOVA adjusted for age, sex, BMI category, teeth number, presence of caries, and periodontal status using adonis function. Permutations were set to 999. The distance matrix of significant taxa was subsequently visualized by PCoA plot. In addition, hierarchical clustering, based on the distance matrix calculated from SNV frequency, was performed, and the resulting dendrogram was visualized. Additionally, we built a strain-level phylogenetic tree by identifying core-genome regions that have high coverage across multiple samples. We performed the call_consensus.py script for the species significantly differed in the SNV frequency comparison with the following parameters; –site_maf 0.01, –site_depth 5, –site_prev 0.90, –sample_depth 10, –sample_cov 0.40, –size_ratio 5.0 as suggested by the MIDAS manual. Subsequently, FastTree^37^ was used to build a phylogenetic tree for the species and we visualized the resulting tree. Within groups strain sharing were measured by the MIDAS script strain_tracking.py, with the parameter of –min_freq 0.90 and –min_reads 10 in the strain_tracking.py id_markers, which identifies SNV that rarely occur in different unrelated samples, and with the default parameter in the strain_tracking.py track_markers, which identifies marker SNV sharing between samples. We defined the SNV sharing cutoff level of 5%, and described the proportion of SNV sharing within groups. Genes contained in representative genomes included in the database were identified and annotated using prokka^38^ version 1.14.0, in addition to the default annotation provided by MIDAS. Specifically, the exact matching of scaffold identifiers, start position, and end position was performed for the MIDAS default database and prokka annotation. Prokka was used with the default parameter. Gene content of each species was compared between current and never smokers by logistic regression models implemented in brglm using the binary table of gene presence of each species adjusted for age, sex, BMI category, teeth number, presence of caries, and periodontal status^39^. Genes present or absent in all the samples, or genes that were present or absent in all the samples in each group were excluded before testing. All statistical tests were two-sided, and a *p*-value, or false discovery rate adjusted *q*-value, of <0.05 was considered statistically significant^40^. All analysis was performed using R or Python. The plots were organized and generated using ape, DECIPHER, dendextend, firatheme, and ggplot2^41–45^.

### Random forest classification

Classification of current and never smokers was achieved using the random forest classifier using the species abundance table, SNV frequency, and gene presence table of each species. The performance was evaluated by area under receiver operator characteristic curve (AUROC). Five fold cross-validation was performed, and the mean and standard deviation of AUROC was calculated. Random forest classification was performed by scikit-learn RandomForestClassifier function, default parameters provided for the function, and a fixed seed number was used^46^.

### Reporting summary

Further information on research design is available in the Nature Research Reporting Summary.

## Supplementary information

Supplementary Information

Reporting Summary

Supplementary Data 1

## Data Availability

All the data supporting the conclusion of this analysis are available on Supplementary Information, the public repository (Supplementary Data, 10.6084/m9.figshare.11611089), or on reasonable request to the corresponding author.

## Code availability

The scripts are available from the corresponding author upon reasonable request.
