# Supplementary material for: Metagenomic analysis of bacterial species in tongue microbiome of current and never smokers
Source: NPJ Biofilms Microbiomes. 2020 Mar 13;6:11. doi: 10.1038/s41522-020-0121-6 (PMC7069950; doi:10.1038/s41522-020-0121-6)
Supplement: Supplementary file 1 — Supplementary Information [file 41522_2020_121_MOESM1_ESM.pdf]

Supplementary Figure 1. The dendrogram based on the consensus core−genome

Supplementary Figure 2. The dendrogram based on the SNV frequency with RefSeq deposited sequences

Supplementary Data Set 1

Sheet Dataset1_1. The result of differential abundance analysis

Sheet Dataset1_2. The number of participants with the sufficient read depth for all the species

Sheet Dataset1_3. The participants' background for significantly differed species in the SNV frequency analysis

Sheet Dataset1_4. The statistical result of SNV frequency comparison between periodontally healthy and suspected of periodontitis participants

Sheet Dataset1_5. The summary of the validation result

Sheet Dataset1_6. The result of strain sharing analysis

Sheet Dataset1_7. The summary of differentially present genes

Sheet Dataset1_8. The summary of the inferred pathway by MinPath

Supplementary Figure 1. The dendrogram based on the consensus core−genome

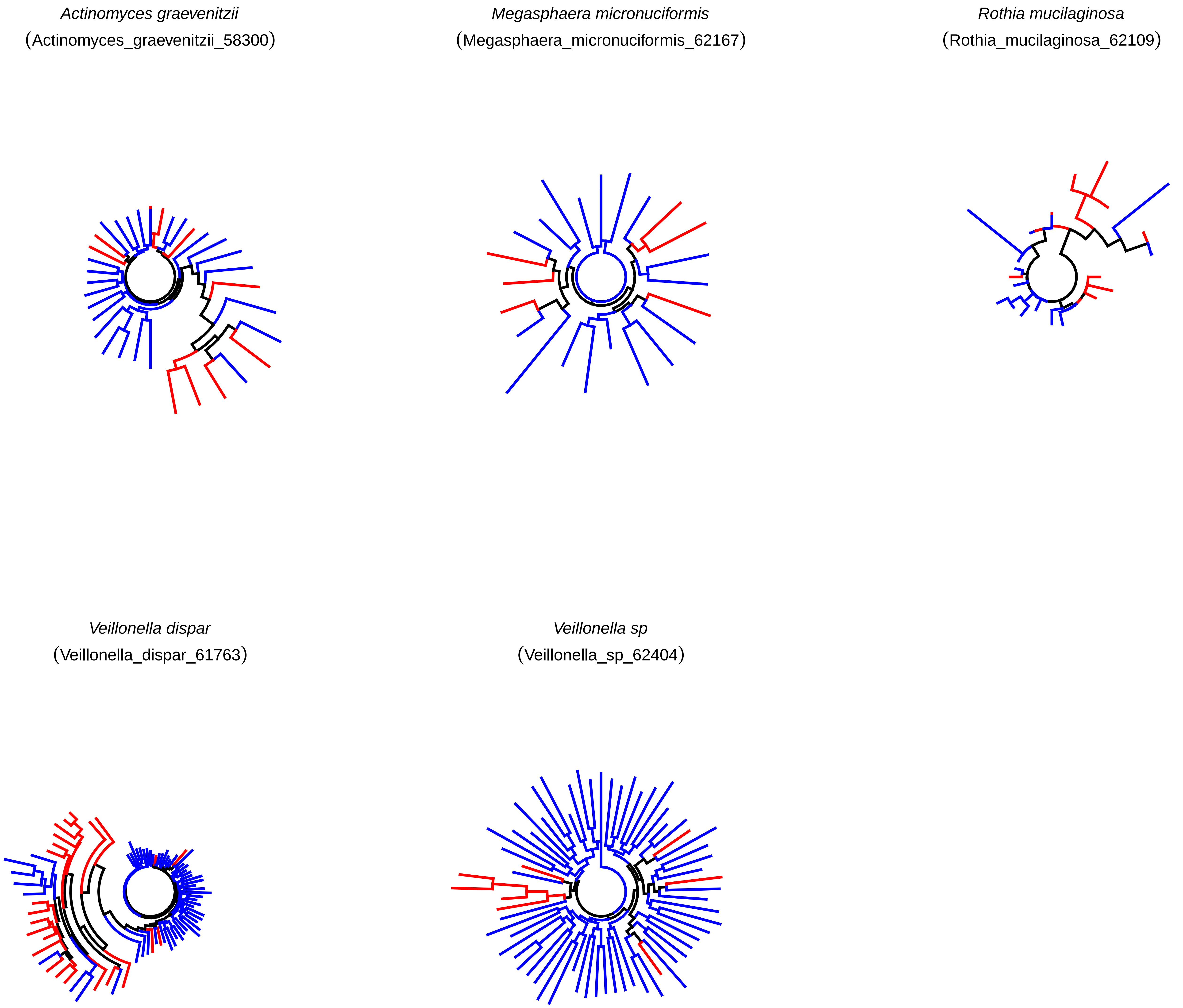

Red branches indicate the leaf of current smokers, and blue branches indicate the leaf of never smokers.

Supplementary Figure 2. The dendrogram based on the SNV frequency with RefSeq deposited sequences

Actinomyces graevenitzii

(Actinomyces_graevenitzii_58300)

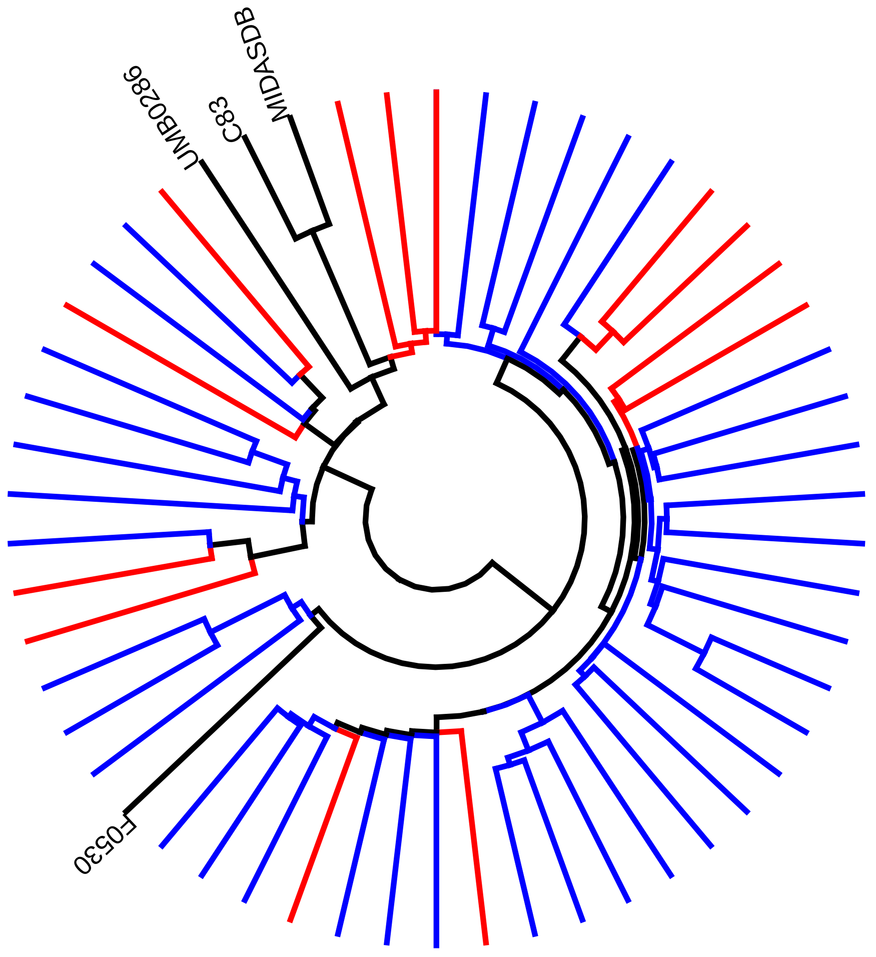

Megasphaera micronuciformis

(Megasphaera_micronuciformis_62167)

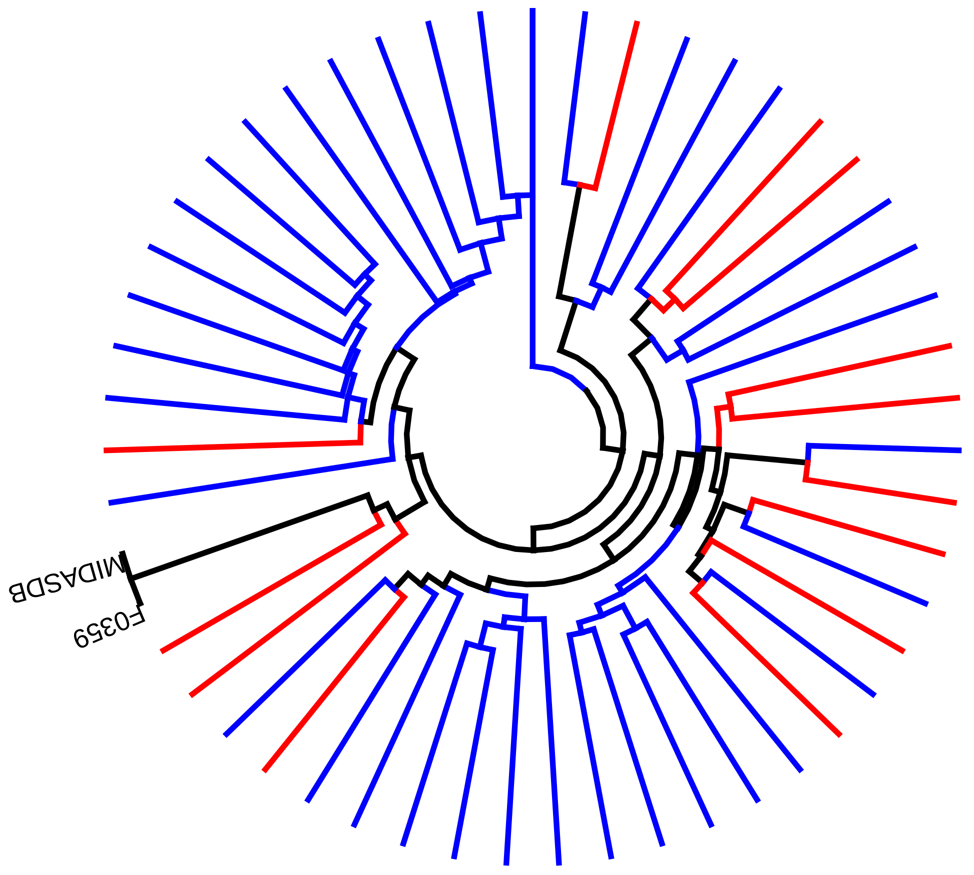

Rothia mucilaginosa

(Rothia_mucilaginosa_62109)

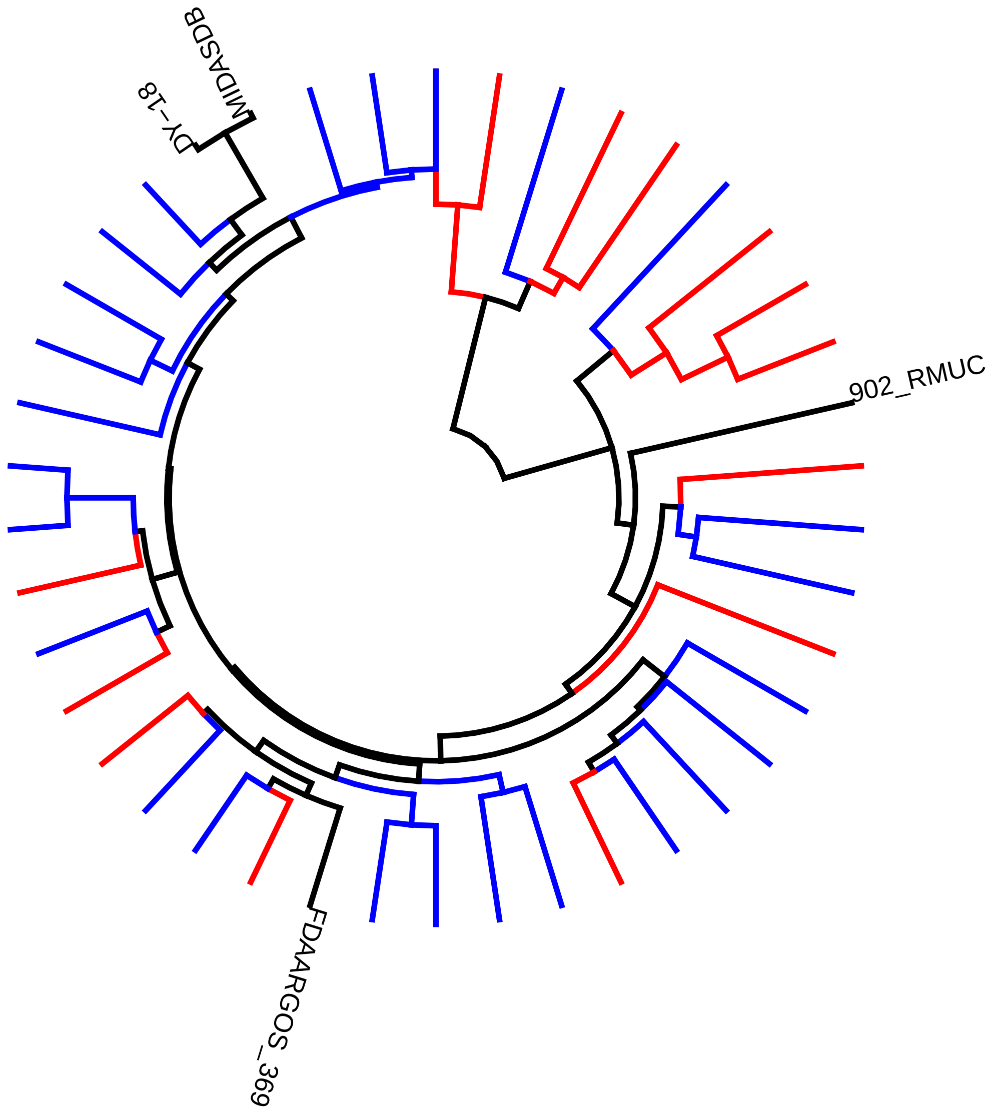

Veillonella dispar

(Veillonella_dispar_61763)

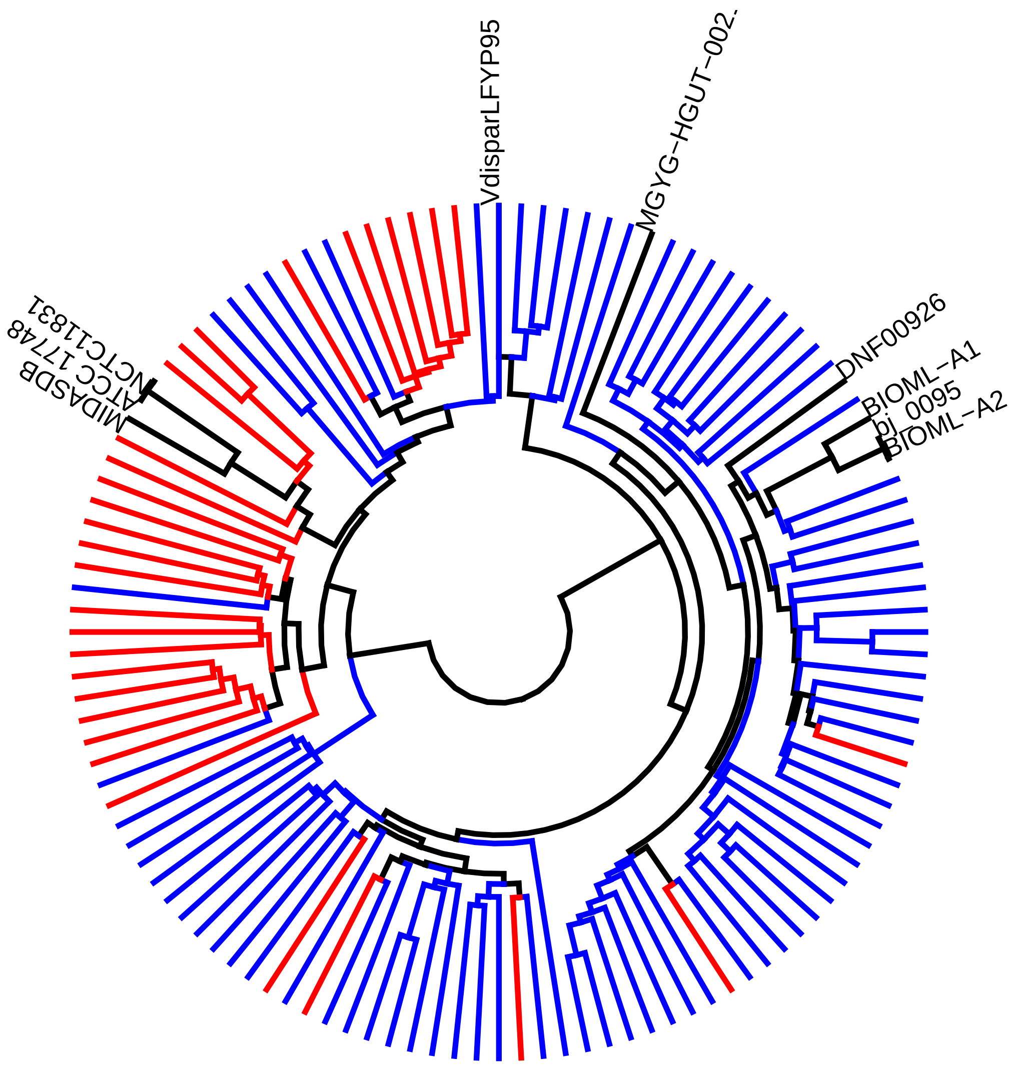

Veillonella sp

(Veillonella_sp_62404)

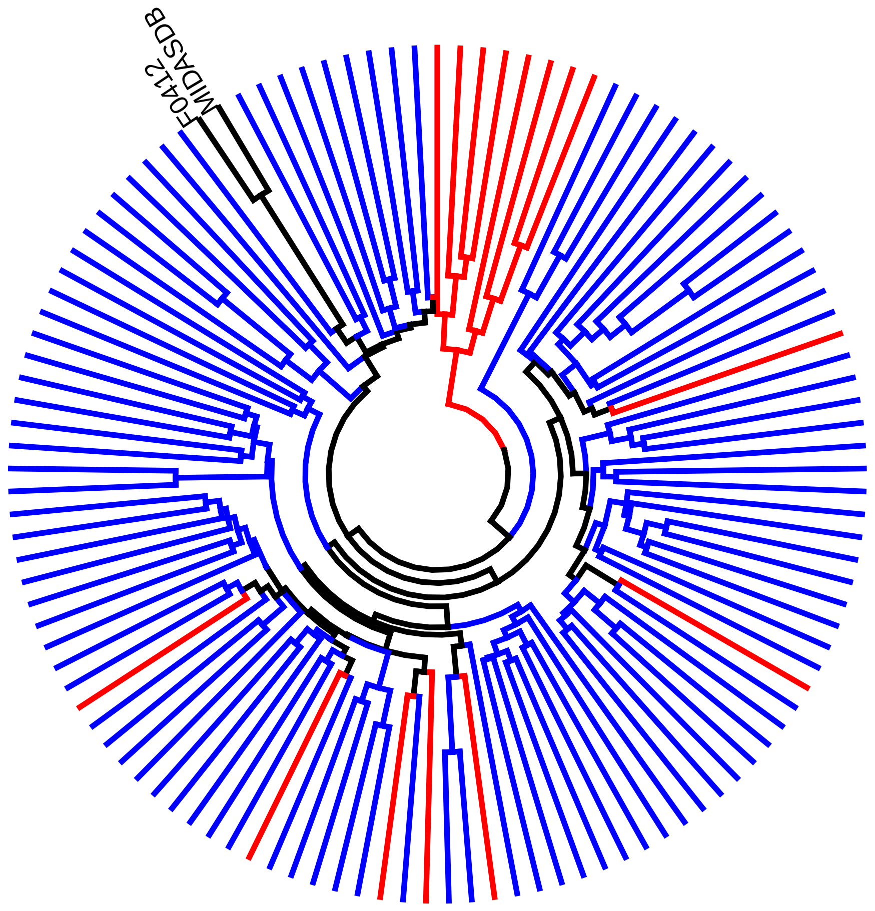

Red branches indicate the leaf of current smokers, and blue branches indicate the leaf of never smokers.
